# Feasibility and Temperature Stability of Low-Complexity Therapeutic Hypothermia for Neonatal Hypoxic–Ischemic Encephalopathy: A Retrospective Case Series

**DOI:** 10.1055/a-2924-5385

**Published:** 2026-08-14

**Authors:** Milagros F. Bermudez-Pelaez, Veronica F. Rojas-Osorio, Cristian M. Matos-Inocente, Marina A. Bustamante-Ordoñez, Maria L. Rospigliosi-Lopez

**Affiliations:** 1 Faculty of Medicine 33216 Universidad Peruana Cayetano HerediaSan Martin de Porres DistrictLima RegionPeru; 2 Faculty of Medicine 471931 Universidad Cientifica del SurLima RegionPeru; 3 Neonatal Intensive Care Unit 280152 Hospital Nacional Cayetano HerediaSan Martin de Porres DistrictLima RegionPeru

**Keywords:** hypoxic–ischemic encephalopathy, therapeutic hypothermia, temperature control, neonate, resource-limited settings, neonatal intensive care, neonatal hypothermia, low-complexity cooling

## Abstract

**Objective** This study aimed to evaluate the feasibility and temperature stability of low-complexity therapeutic hypothermia (TH) in neonates with moderate-to-severe hypoxic–ischemic encephalopathy (HIE).

**Study Design** Retrospective case series of neonates born at ≥36 weeks of gestation with moderate-to-severe HIE treated with low-complexity TH in a tertiary-level hospital in Peru (2018–2024). Variables included timing of initiation, achievement and maintenance of the target core temperature, duration of hypothermia, and clinical outcomes. Descriptive statistics were used.

**Results** A total of 13 neonates were included (77% male; median gestational age 38 weeks). TH was initiated at a median of 103 min after birth. Within the first 6 hours, 84.6% reached 34°C; however, only 31% maintained a stable core temperature within the target range (33.5–34.5°C) throughout treatment. Median duration of hypothermia was 75 hours. Complications occurred in 23.1% of cases, and overall mortality was 15.4%.

**Conclusion** Low-complexity TH is feasible and allows timely initiation within the neuroprotective window. However, maintaining stable therapeutic temperatures remains a major challenge, which may compromise its potential neuroprotective effectiveness.

## Introduction

Hypoxic–ischemic encephalopathy (HIE) is a major cause of neonatal morbidity and mortality worldwide, resulting from perinatal asphyxia and leading to significant neurological injury. 1 Following the initial hypoxic insult, a latent phase of approximately 6 hours provides a critical window for intervention before secondary energy failure and neuronal death occur. 1 Clinically, HIE presents with a spectrum of neurological abnormalities 2 3 and is commonly classified using the Sarnat staging system. 4 5

The incidence of HIE ranges from 1 to 8 per 1,000 live births in high-income countries and up to 26 per 1,000 in low- and middle-income settings. 6 Moderate-to-severe HIE is associated with a high risk of death and long-term neurodevelopmental impairment. 7

Therapeutic hypothermia is the standard of care for neonates with moderate-to-severe HIE. 8 It should be initiated within the first 6 hours of life and maintained for 72 hours at a target temperature of 33–35°C. 8 In high-resource settings, servo-controlled cooling devices enable precise temperature regulation and improve clinical outcomes. 9 However, these technologies are often unavailable in resource-limited settings.

As a result, low-complexity cooling methods—such as passive cooling and the use of gel packs—are commonly employed as accessible alternatives. 10 While some studies suggest benefits in reducing mortality, 9 these approaches rely on manual adjustments and may result in suboptimal temperature control. 10 11 Maintaining stable therapeutic temperatures remains a major challenge, which may compromise the neuroprotective effect of hypothermia. 11

However, data on real-world implementation and temperature control using low-complexity methods remain limited, particularly in low- and middle-income settings. Understanding their performance under routine clinical conditions is essential to optimize neonatal care where advanced technologies are not available.

Therefore, this study aimed to evaluate the feasibility, temperature stability, and short-term clinical outcomes in neonates with moderate-to-severe HIE treated with low-complexity therapeutic hypothermia in a tertiary-level hospital in Peru.

## Materials and Methods

### Study Design and Population

We conducted a retrospective case series of neonates with HIE treated with therapeutic hypothermia. Medical records were reviewed to extract relevant clinical data. Eligible patients were neonates born at ≥36 weeks of gestation diagnosed with moderate-to-severe HIE who received low-complexity therapeutic hypothermia in the neonatal intensive care unit of a tertiary-level hospital in Peru between 2018 and 2024.

All included neonates initiated hypothermia within the first 6 hours of life. Newborns transferred from another facility after birth were excluded.

This study was approved by the Institutional Ethics Committee of Universidad Peruana Cayetano Heredia (SIDISI code: 211986).

### Data Collection

Maternal, perinatal, and neonatal characteristics were obtained from medical records. The severity of HIE was assessed using either the Sarnat classification or the Thompson score, according to the attending physician’s clinical judgment.

Variables collected included time from birth to initiation of hypothermia, time to reach a core temperature of 34°C, duration of maintenance of the target core temperature, total duration of hypothermia, rewarming time, length of hospitalization, and the use of supportive therapies, including sedation, ventilatory support, antibiotics, inotropes, and anticonvulsants. Complications occurring during treatment were also recorded.

### Eligibility Criteria for Therapeutic Hypothermia

Eligibility criteria for therapeutic hypothermia included a Thompson score ≥7 or a diagnosis of moderate-to-severe HIE based on the Sarnat classification, in addition to biochemical and clinical parameters such as umbilical cord pH or blood gas pH ≤ 7.00 within the first hour of life, base excess ≤−12 mEq/L, and a 10-min Apgar score ≤5.

### Therapeutic Hypothermia Protocol

Following the diagnosis of moderate-to-severe HIE, external heat sources were discontinued, and passive cooling was initiated immediately while vascular access and supportive care were established. Continuous core temperature monitoring was performed using a rectal temperature probe connected to an iMEC15 monitor (Mindray, Shenzhen, China). The probe remained in place throughout the 72-hour cooling period and until completion of rewarming. Temperature alarms were set manually at 33.5°C (lower limit) and 34.5°C (upper limit).

Cooling was achieved using five reusable 500-g gel packs routinely employed for vaccine cold-chain transport. Each gel pack was wrapped in a cotton cloth to prevent direct skin contact. Initially, three gel packs were positioned around the head (one superiorly and one on each side) and two were placed along the lateral aspects of the torso, maintaining a distance of approximately 10 cm from the neonate.

Core temperature was monitored continuously by neonatal intensive care unit (NICU) nursing staff, with a nurse-to-neonate ratio of approximately 1:2. Temperature measurements and nursing interventions were documented using a standardized bedside nursing monitoring form ( **Supplementary Fig. S1** , available in the online version only). Gel pack adjustments were performed manually according to the neonate’s core temperature. When the core temperature exceeded 34.5°C, gel packs were repositioned or replaced to restore the target therapeutic temperature. If the core temperature decreased below 33.5°C, one gel pack was removed until the target therapeutic range was restored. The attending neonatologist was consulted whenever unexpected clinical events or difficulties in temperature management occurred.

Gel packs were not replaced at predetermined intervals; instead, they were exchanged whenever they lost their cooling capacity or when the neonate’s core temperature exceeded the therapeutic target.

Therapeutic hypothermia was maintained for 72 hours, followed by gradual manual rewarming over approximately 6–12 hours. During rewarming, all gel packs were removed, and the Giraffe incubator was turned on. Rewarming was performed at a target rate of approximately 0.5°C/h with continuous core temperature monitoring. If the core temperature increased more rapidly than the target rate, a gel pack was temporarily reapplied to prevent temperature overshoot. Core temperature monitoring continued until it reached ≥36.5°C, after which routine axillary temperature monitoring was resumed. Neurological assessment was performed using either Sarnat staging or the Thompson score according to clinician preference and was confirmed by a pediatric neurologist in all cases.

### Statistical Analysis

Descriptive statistics were used. Continuous variables are presented as medians and interquartile ranges (IQRs), and categorical variables as frequencies and percentages.

## Results

A total of 13 neonates treated between 2018 and 2024 were included. Most were male (76.9%), with a median gestational age of 38 weeks (IQR: 37–39; range 36–41), and a median birth weight of 3,565 g (IQR: 3,020–3,640; range: 2,250–4,380). All neonates required resuscitation at birth. Baseline neonatal, maternal, and perinatal characteristics are summarized in Tables 1 2 3 .

**Table 1:** Characteristics of the newborns.

Characteristics of newborns		Patients												
		1	2	3	4	5	6	7	8	9	10	11	12	13
Sex		M	M	M	M	M	M	M	F	M	F	F	M	M
Gestational age (w)		37	36	39	36	37	38	38	39	38	41	39	41	40
Weight at birth (g)		3,000	3,149	3,585	2,250	2,888	4,320	3,480	3,565	3,590	3,640	3,020	4,380	3,820
APGAR	1 min	6	0	5	4	4	0	4	4	1	4	5	1	3
	5 min	8	1	8	8	7	0	6	8	4	5	7	4	8
	10 min	ND	5	ND	ND	7	1	ND	ND	6	5	9	5	ND
Neonatal resuscitation		Yes	Yes	Yes	Yes	Yes	Yes	Yes	Yes	Yes	Yes	Yes	Yes	Yes
Endotracheal tube placement		No	Yes	No	No	No	Yes	Yes	No	No	No	No	Yes	No
ABG within the first hour of life		ND	7.022	7.13	7.034	ND	7.04	ND	ND	ND	ND	7.012	ND	ND
Umbilical cord ABG		6.89	ND	ND	ND	7.00	ND	7.00	7.00	6.9	6.95	ND	6.82	6.8
Base deficit ≥−12 mEq/L		−14	−17	−16	−18	−15	−20	−13	−18	−16	−24	−20	−28	−22

Abbreviations: ABG, arterial blood gas; APGAR, score at 1, 5, and 10 min based on appearance (skin color), pulse (heart rate), grimace (reflex response), activity (muscle tone), and respiration (breathing effort); F, female; M, male; ND, not documented.

**Table 2:** Maternal characteristics.

Maternal characteristics	Patients												
	1	2	3	4	5	6	7	8	9	10	11	12	13
Maternal age	20	30	30	33	25	27	34	28	40	35	25	24	32
Maternal comorbidity	ND	ND	ND	ND	PBC consumption	ND	Umbilical cord prolapse	UTI, PROM	No	No	UTI	PROM	UTI
GTPAL	G1T0P0A0L0	G2T1P0A0L1	G2T1P0A0L1	G6T3P1A1L4	G6T5P0A0L5	G4T2P0A2L1	G4T2P0A1L2	G1T0P0A0L0	G7T6P0A0L6	G2T1P1A0L1	G1T0P0A0L0	G5T2P0A2L2	G2T1P0A0L1

Abbreviations: GTPAL, gravida (total pregnancies), term births (≥37 weeks), preterm births (<37 weeks), abortions (<20 weeks), living children; ND, not documented; PBC consumption, psychoactive substance consumption during pregnancy; PROM, prelabor rupture of membranes; UTI, urinary tract infection.

**Table 3:** Characteristics of childbirth.

Characteristics of childbirth	Patients												
	1	2	3	4	5	6	7	8	9	10	11	12	13
Acute fetal distress	No	Yes	No	No	No	Yes	Yes	No	No	Yes	Yes	Yes	Yes
Sentinel event	ND	Fetal bradycardia	Fetal bradycardia	Placental abruption	ND	Fetal bradycardia	Fetal bradycardia, umbilical cord prolapse	ND	ND	Fetal bradycardia	Fetal bradycardia	Fetal bradycardia	ND
Type of delivery	SVD	Cesarean section without labor	SVD	Cesarean section with labor	SVD	SVD	Cesarean section with labor	SVD	SVD	Cesarean section without labor	Cesarean section without labor	SVD	Cesarean section without labor

Abbreviations: ND, not documented; SVD, spontaneous vaginal delivery.

Sarnat classification was used in four neonates (30.8%), whereas the Thompson score was used in nine (69.2%), following its introduction into clinical practice at our institution in 2021. HIE was classified as moderate in 10 neonates (76.9%) and severe in 3 (23.1%; Table 4 ).

**Table 4:** Management characteristics.

Management characteristics	Patients												
	1	2	3	4	5	6	7	8	9	10	11	12	13
Hypoxic–ischemic encephalopathy classification	Moderate	Severe	Moderate	Moderate	Moderate	Severe	Moderate	Moderate	Moderate	Sarnat II	Sarnat II–III	Sarnat II	Sarnat II
Time of life at onset of hypothermia (h)	2:22	1:30	2:29	1:43	0:56	1:54	1:05	1:07	2:13	1:13	3:15	4:00	1:03
Time in which 35°C was reached	9:27	1:36	2:37	4:08	1:00	1:56	2:15	5:37	3:13	9:43	5:17	1:15	4:03
Time in which 34°C was reached	11:27	1:38	2:40	5:53	1:02	2:00	3:15	6:02	5:13	11:43	6:45	1:15	3:33
Hypothermia was maintained at a temperature of 34°C	No	Yes	No	No	Yes	No	No	No	No	Yes	No	No	Yes
Duration of hypothermia (h)	Died	77:20:00	73:30:00	77:25:00	75:20:00	75:00:00	75:00:00	73:25:00	76:00:00	65:30:00	71:45:00	72:15:00	76:43:00
Reheating time (h)	Died	13:03	8	15	9:05	12	10	5	7	7:20	ND	8	9
Duration of hospitalization (d)	2 (died)	13	4 (died)	10	15	60	12	16	13	10	14	17	15
Use of sedation	Yes	Yes	Yes	Yes	Yes	Yes	Yes	Yes	Yes	Yes	Yes	Yes	Yes
Use of ventilatory support	Yes	Yes	Yes	Yes	Yes	Yes	Yes	Yes	Yes	Yes	Yes	Yes	Yes
Use of antibiotics	Yes	Yes	Yes	Yes	Yes	Yes	No	Yes	Yes	Yes	Yes	No	No
Use of inotropes	Yes	Yes	Yes	No	No	Yes	Yes	No	No	No	Yes	No	No
Use of anticonvulsant medication	No	No	No	No	No	No	No	No	No	No	Yes	No	Yes
Complications during handling	No	No	No	No	No	No	No	Pneumothorax, meconium aspiration syndrome	Coagulation disorder due to asphyxiation	No	Tonic-clonic seizures	Erb-Duchenne palsy	No

Abbreviation: ND, not documented.

**Fig. 1:**
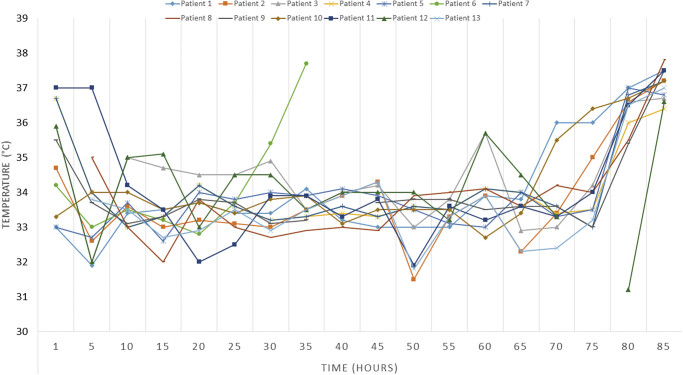
Temperature trajectory in neonates receiving therapeutic hypothermia for hypoxic–ischemic encephalopathy. Core body temperature (°C) over time (hours) in 13 neonates undergoing low-complexity therapeutic hypothermia. The phases of induction (cooling), maintenance, and rewarming are illustrated. The target therapeutic range (33–35°C) is indicated. Interindividual variability highlights challenges in achieving and maintaining temperature stability.

Therapeutic hypothermia was initiated at a median of 103 hours after birth (IQR: 66–146; range 56–240). The median time to reach 35 and 34°C was 3 hours 13 minutes and 3 hours 33 minutes, respectively. Within the first 6 hours of life, 84.6% of neonates reached 34°C and 69.2% reached 35°C. Although the majority of neonates reached the target core temperature within the first 6 hours of life, only 31% maintained the target core temperature of 34°C throughout the treatment period ( Table 4 ). The median duration of hypothermia was 75 hours (IQR: 72.8–76.4; range: 65.5–77.4). The duration of maintenance of the target core temperature ranged from 51 to 77 hours. Rewarming was performed over a median of 9 hours (IQR: 7.3–12; range: 5–15). One neonate underwent therapeutic hypothermia for only 24 hours due to clinical deterioration and subsequently died during hospitalization ( Fig. 1 ).

The median length of hospitalization was 13 days (IQR: 10–15.5). All neonates required sedation and ventilatory support, while antibiotics, inotropes, and anticonvulsants were administered in 76.9, 46.2, and 15.4% of cases, respectively ( Table 4 ).

Complications occurred in 23.1% of neonates and included pneumothorax, coagulation disorders, and tonic–clonic seizures. Overall mortality was 15.4%.

## Discussion

In this case series, low-complexity therapeutic hypothermia was feasible and allowed timely initiation within the recommended neuroprotective window. 12 13 All included neonates met established international criteria for therapeutic hypothermia, supporting appropriate patient selection according to current clinical guidelines. 13

Neurological assessment was performed using both Sarnat staging and the Thompson score, reflecting changes in clinical practice over time. Although both tools have been validated, their heterogeneous use may have introduced variability in the classification of HIE severity and limited comparability with other studies. 14 15 16 These findings highlight the importance of implementing a standardized neurological assessment protocol to improve diagnostic consistency and facilitate comparisons across settings.

A key finding of this study is the limited ability of low-complexity cooling to maintain stable core temperatures within the therapeutic range. Although most neonates reached the therapeutic range within the first 6 hours of life, only 31% maintained a stable target core temperature of 34°C throughout the treatment period. Temperature instability was frequent, with the majority of neonates experiencing deviations from the target therapeutic range. 12

This variability is likely multifactorial. The inconsistent thermal capacity of gel packs, environmental conditions, individual neonatal physiology, and the need for frequent manual adjustments may all contribute. In addition, the lack of automated temperature regulation systems may further compromise stability. These findings are consistent with prior studies from resource-limited settings, where suboptimal outcomes with therapeutic hypothermia have been associated with challenges in maintaining precise temperature control. 14 17 18 19

Importantly, although most neonates remained within the broader therapeutic range (33–35°C) for more than 50 hours, only one achieved continuous maintenance of the target core temperature throughout the recommended 72-hour cooling period. This is clinically relevant, as consistent temperature control is considered essential to maximize the neuroprotective effects of hypothermia. 13 Suboptimal temperature regulation may reduce the potential neuroprotective benefit of hypothermia and increase the risk of adverse physiological effects, including hemodynamic instability and metabolic disturbances. Despite these limitations, the observed mortality rate (15.4%) was within the range reported in studies using advanced cooling technologies. 14 15 20 While this finding should be interpreted cautiously given the small sample size, it suggests that low-complexity hypothermia may represent a feasible therapeutic option in settings where advanced systems are not available.

The high rate of antibiotic use observed in this cohort likely reflects a preventive approach in critically ill neonates undergoing invasive interventions. 21 However, this practice underscores the need for antimicrobial stewardship strategies, given the potential long-term impact on the developing microbiome. 22

All neonates required sedation and ventilatory support, reflecting the severity of illness. Sedation is an essential component of therapeutic hypothermia, as it reduces metabolic demand and prevents shivering, thereby supporting neuroprotection. 23 24 25 26 Similarly, the frequent need for mechanical ventilation is consistent with previous reports in neonates with moderate-to-severe HIE. 27 28 29 The rewarming phase was conducted according to international recommendations, with gradual temperature increase of approximately 0.5°C/h. 30 Although often underemphasized, this phase is critical, as rapid or poorly controlled rewarming may negatively impact neurological outcomes. 31 32

This study has several limitations, including its small sample size, single-center design, and lack of long-term neurodevelopmental follow-up. Additionally, variability in neurological assessment methods and temperature monitoring may have influenced the results. Despite these limitations, this study provides valuable evidence from a resource-limited setting, demonstrating that low-complexity therapeutic hypothermia can be implemented. However, its potential neuroprotective benefit may be constrained by challenges in maintaining stable target temperatures. 10

Future efforts should focus on protocol standardization, improved training, and the development of cost-effective technologies to enhance temperature control. These strategies are essential to maximize the safety and neuroprotective effectiveness of therapeutic hypothermia in resource-limited settings. 14 18 19

## Data Availability

The final study dataset will be available through a secure and supervised data enclave managed by the Research Unit of the Neonatal Intensive Care Department at Hospital Nacional Cayetano Heredia. The dataset will include anonymized clinical information from all neonates included in the study between 2018 and 2024. Access will be restricted to registered investigators who submit a specific research proposal and analysis plan and sign a data use agreement in compliance with local ethical and institutional guidelines. Individual requests will be reviewed to safeguard the intellectual property of the study team and to prevent overlap with ongoing or planned analyses by the original investigators.

## Supplementary Material

Ergänzendes Material Supplementary Material
