# Supplementary material for: Feasibility and Temperature Stability of Low-Complexity Therapeutic Hypothermia for Neonatal Hypoxic–Ischemic Encephalopathy: A Retrospective Case Series
Source: AJP Rep. 2026 Aug 14;16(3):e134–40. doi: 10.1055/a-2924-5385 (PMC13476106; doi:10.1055/a-2924-5385)
Supplement: Supplementary file 1 [file 10-1055-a-2924-5385_29384795.pdf]

Thieme

REGISTRO DEL MONITOREO DE ENFERMERÍA - NOTAS DE ENFERMERÍA
UNIDAD DE CUIDADOS INTENSIVOS NEONATALES
I. VALORACIÓN

NOMBRES Y APELLIDOS:	DIAGNOSTICO MÉDICO:	FECHA DE NACIMIENTO:	PESO NACIMIENTO:
H.C.		FECHA DE INGRESO UCI	PESO INGRESO:
GS RN / SEXO F		EDAD GESTACIONAL:	PESO ACTUAL:
GS MADRE / SEXO M		EDAD CORREGIDA:	VARIACIÓN DE PESO:
FECHA	TAMIZAJE ROP:	DÍAS VIDA: DÍAS EN UCI:	TALLA: PC:

Sección	HORAS	8	9	10	11	12	13	14	15	16	17	18	19	BH 12 HRS	20	21	22	23	24	1	2	3	4	5	6	7	BH 12 HRS	BH 24 HRS
MONITOREO HEMODINÁMICO	T° AXILAR- T° RECTAL																											
	T° INCUB. / HUMED																											
	FRECUENCIA CARDIACA																											
	CARACTERISTICAS																											
	FRECUENCIA RESPIRATORIA																											
	CARACTERISTICAS																											
	PAS / PAD																											
	PAM																											
	SAT O2 PREDUCTAL																											
	SAT O2 POSTDUCTAL																											
	LLENADO CAP. / IP																											
	CARACTERISTICAS DE LA PIEL																											
MONITOREO RESPIRATORIO VENTILATORIO	MODO																											
	IO / CO2 EXH																											
	PIM																											
	PEEP																											
	FR/HERTZIOS																											
	PAM - MAP																											
	FLUJO																											
	SENSIBILIDAD																											
	FIO2																											
	VOL.GARANTIZA-VTHF																											
	COMPLIANCE /DCO2																											
	RESIST. / AMPLITUD																											
	TUBO/ALTURA																											
	ADM.SURFACTAN																											
MONITOREO NEUROLÓGICO	PUPILA - TAMAÑO																											
	PUPILA - RECTIVIDAD																											
	FONTANEL / CARACT.																											
	ESCALA DE DOLOR																											
	POSICIÓN																											
	HORAS	8	9	10	11	12	13	14	15	16	17	18	19	BH 12 HRS	20	21	22	23	24	1	2	3	4	5	6	7	BH 12 HRS	BH 24 HRS
	PERIMETRO ABDOMINAL																											
... HÍDRICO ELECTROLÍTICO RENAL - GASTROINTESTINAL / INGRESOS / ENTERAL	NPO																											
	CALOSTRO - NEM																											
	LM																											
	LM +																											
	FORMULA																											
	FORMULA +																											
	LMD																											
	SNY																											
	SOG																										TOTAL:	
INGRESOS / PARENTERAL																												
	SOG																											
																											TOTAL:	
EGRESOS	SNY																											
	VÓMITO / CARACTERISTICAS																											
	RESID. GASTRIC. CARACT.																											
	DEPOSICIÓN - CARACTERISTICAS																											
	OSTOMÍA - CARACTERISTICAS																											
	ORINA - CARACTERISTICAS																											
	FLUJO URINARIO																											
	DRENAJE (1)																											
	MUESTRA DE SANGRE																											
	GLICEMIA																											
	FOTOTERAPIA																											

GRADO DE DEPENDENCIA

	I D	I N	II D	II N	III D	III N
PRIORIDAD DE ATENCIÓN						
PUNTAJE NEMS						

GRADO DE DEPENDENCIA

DISPOSITIVOS	LUGAR INSERCIÓN	FECHA DE ENSERCIÓN	DÍAS DE USO	FECHA DE RETIRO	FECHA DE CURACIÓN
CVP					
CVP					
CVC					
CAC					
CVC					
PICC 1					
ING (cms)					
QUEDA (cms)					
PICC 2					
ING (cms)					
QUEDA (cms)					

PERFIL DE DOLOR PARA NEONATOS PREMATUROS (PIPP)

INDICADOR	0	1	2	3
EDAD GESTACIONAL	36 semanas y más	32 semanas a 35 semanas	28 semanas y 6 días	28 semanas y menos
COMPORTAMIENTO 15 SEG	Activo / despierto ojos abiertos, movimientos faciales	quieto/despierto ojos abiertos no movimientos fasciales	Activo/dormido ojos cerrados movimientos fasciales	Activo/dormido ojos cerrados movimientos
FRECUENCIA CARDIACA (max) 30 seg	Aumento de 0 a 4 latidos / minuto	Aumento de 5 a 14 latidos / minuto	Aumento de 15 a 24 latidos / minutos	Aumento de 25 latidos/minuto
SATURACIÓN DE OXIGENO (max) 30 seg	0 a 2.4% disminución	2.5 a 4.9% disminución	5.0 a 7.4% disminución	7.5% o mas disminución
ENTRECEJO FRUNCIDO 30 seg	Nunca 0-3 segundos	Mínimo > 3 - 12 segundos	Moderado > 12 - 21 segundos	Máximo > 2 1 - 30 segundos
APRIETA LOS OJOS 30 SEG	Nunca 0-3 segundos	Mínimo > 3 - 12 segundos	Moderado > 12 - 21 segundos	Máximo > 2 1 - 30 segundos
ARRUGA NASOLABIAL 30 SEG	Nunca 0-3 segundos	Mínimo > 3 - 12 segundos	Moderado > 12 - 21 segundos	Máximo > 2 1 - 30 segundos

Puntaje 6 o menor	Mínima o sin dolor
7 a 12	Dolor apacible (use intervenciones farmacológicas)
> 12	Dolor moderado (use intervenciones farmacológicas y no farmacológicas)

PERFIL DEL DOLOR PARA NEONATOS ESCALA CRIES

PARÁMETROS	0 puntos	1 punto	2 puntos
Llanto	No	Agudo consolable	inconsolable
FIO2 para Sat O2 > 95	0.21	< 0 = 03	> 0.3
FC y TA	< 0 = basal	> o > 20% basal	> o > 20% basal
Expresión Facial	Normal	Muecas	Muecas / gemido
Períodos de sueño	Normales	Despierto muy frecuente	Despierto constantemente

PUNTAJE

0 a 6 :	Sin Dolor I Dolor leve
7 a 12 :	Dolor Moderado
Mayor de 12 :	Dolor Intenso

FECHA	ANÁLISIS DE GASOMETRÍA	FECHA	RESULTADOS DE LABORATORIO

BALANCE HÍDRICO 12 Hrs

INGRESO TOTAL	EGRESO TOTAL - EGRESO	EGRESO TOTAL - PI	TOTAL
FIRMA Y SELLO			

BALANCE HÍDRICO 24 Hrs

INGRESO TOTAL	EGRESO TOTAL - EGRESO	EGRESO TOTAL - PI	TOTAL
FIRMA Y SELLO			

CONOCIMIENTO

PROCESO DE ATENCIÓN DE ENFERMERÍA / NOTAS DE ENFERMERÍA
UNIDAD DE CUIDADOS INTENSIVOS NEONATALES

I VALORACIÓN POR DOMINIOS

DOMINIO 1: PROMOCIÓN DE SALUD
CLASE 1: Toma conciencia de salud

	D	N
Conocimiento de padres sobre cuidado del neonato	SI / NO	SI / NO
Inicia extracción de calostro	SI / NO	SI / NO
Se extrae Leche Materna	SI / NO	SI / NO

DOMINIO 2: NUTRICIÓN
CLASE 1: Ingestión 2 Digestión

Succión / Deglución	D	N
Buena - Regular		
Mala - Ausente		
Coordinado no sostenido		

Tolerancia Oral:

Buena		
Mala		

RHA:

Presentes / Aumentados		
Disminuido / Ausente		

Abdomen:

Blando / Depresible		
Distendido		
Tenso		
Doloroso		

CLASE 5: Hidratación

Mucosa Oral:	D	N
Húmeda		
Seca		
Lesiones ______		

Piel:

Indemne		
Edema		
Empastado		
Lesiones ______		

DOMINIO 3: ELIMINACIÓN
CLASE 1: Sistema Urinario

Diuresis:	D	N
Espontáneo		
Globo Vesical		
Cateterismo		

Flujo:

Normal		
Poliuria		
Oliguria		
Anuria		

CLASE 2: Sistema Gastrointestinal

Deposición:	D	N
Ano		
Espontáneo		
Estimulación / Enema		
Ostomía		

CLASE 4: Sistema Pulmonar

Simetría Toráxica:	D	N
Simétrico		
Asimétrico		
Ruidos Agregados:		
Crepitantes		
Roncantes		
Sibilantes		
Estridor		

Secreciones Bronquiales:

Blanquecinas		
Verde meconial		
Densa / Clara		
Sanguinolenta		
Fluida		
Tapones		

Drenaje Toráxico:

Succión Continua		
Succión Intermitente		
Presión en mmHg		

DOMINIO 4: ACTIVIDAD REPOSO
ESCALA BRAZELTON
CLASE 1: Reposo y sueño

Estado Sueño / Vigilia:	D	N
Sueño profundo		
Sueño en calma		
Alerta en calma		
Irritable		

Regulación del Estado:

Se consuela sólo		
Se interviene		

Organización del Estado:

Irritable		
Agitado		
Llanto		
En calma		
Letárgico		

ESCALA ALS: ADAPTACIÓN
Sistema Nervioso Autónomo:

Tremores		
Sobresalto		

Sistema Motor:

Conservado		
Hipotónico		
Hipertónico		
Flácido		
Mov. Asimétricos		

Social/Interactivo:

Activo		
Reactivo		
Hipoactivo		

CLASE 4: Respuesta Cardiovascular

Activ. Circulatoria:	D	N
Prec. Normodinámico		
Prec. Hiperdinámico		
Soplo		
Pulso Palmar		

DOMINIO 5: PERCEPCIÓN/COGNICIÓN
CLASE 1: Sensación / Percepción

Convulsión:	D	N
Sutil		
Tónico		
Clónico		
Mioclonía		
Coma		

Reflejos:

Moro Completo		
Moro Incompleto		
Búsqueda		
Succión		
Presión Palmar		
Presión Plantar		
Babinski		

DOMINIO 6: AUTOPERCEPCIÓN
CLASE 1: Autoconcepto

Participación Madre:	D	N
Participa del cuidado		
No participa		
Ausente		
Madre Hospitalizada	D	N
(SI) Nº Cama ______		

Visita del Padre:

Siempre		
A veces		
Nunca		

TEST SILVERMAN ANDERSON:

SCORE	D	N
Sin dificultad respiratoria (0)		
Con dificultad respiratoria leve (1 a 3)		
Con dificultad respiratoria moderada (4 a 6)		
Con dificultad respiratoria severa (7 a 10)		

DOMINIO 7: ROL / RELACIONES
CLASE 2: Relaciones Familiares

Fuentes de apoyo:	D	N
Abuelos maternos		
Abuelos paternos		
Otros		

Interacción:

Lo cargan/canguro		
Le hablan/cantan		
Lo tocan		

DOMINIO 8: SEXUALIDAD

Definido		
Ambiguo		

DOMINIO 9: AFRONTAMIENTO / TOLERANCIA ESTRES EN LOS PADRES
CLASE 2: Respuesta Afrontamiento

Conducta de Padres:	D	N
Preocupados		
Ansiosos		
Tristes		
Rechazo		
Temor		
Ausente		

DOMINIO 10: PRINCIPIOS VITALES

Religión de los padres ______		
Bautizado	SI	NO

DOMINIO 11: SEGURIDAD Y PROTECCIÓN

	D	N
CLASE 1: Infección	SI	NO

CLASE 2: Protección Física

Exposición a:	D	N
Procedimientos invasivos	SI / NO	SI / NO

CLASE 4: Peligros Ambientales

Peligros:	D	N
Uso de 02		
Uso fototerapia		
Exposición ocular		
Uso de dispositivo nasal		
Fracturas		

ESCALA VISUAL DE ERITEMA DE PAÑAL (EVE):

No Eritema (0)		
Poco eritema casi imperceptible (1)		
Eritema Moderado (piel rosada) (2)		
Eritema Intensa(piel roja ó púrpura) (3)		
Piel rota o abrasión (superficial) (4)		

PIEL PERIOSTOMAL:

Piel eritematosa		
Lesión Pequeña aislada		
Lesión extensa		
No eritema		

CLASE 6: Termorregulación

Requiere:	D	N
Incubadora / Servocuna		
Incubadora y Humedad		
Cuna		
Hipotermia Inducida		

DOMINIO 12: CONFORT
CLASE 1: CONFORT AMBIENTAL

Manejo del dolor	D	N
Cambio de posición		
Manipulación mínima		
Interv. farmacológico		
Interv. no farmacológica		

CLASE 2: Confort Físico

Posición:	D	N
En flexión		
Hacia línea media		
Deja llevarse mano a la boca		
Nido contención		

DOMINIO 13: CRECIMIENTO Y DESARROLLO

Vacunas	SI	NO

FISIOTERAPIA

Física/ Sensorial / Orofacial		

NOTAS DE ENFERMERÍA

FIRMA Y SELLO

NOTAS DE ENFERMERÍA

FIRMA Y SELLO

II DIAGNOSTICOS DE ENFERMEROS

COD. NANDA	TURNO DIURNO	COD. NANDA	TURNO NOCTURNO

III PLANIFICACIÓN DE ENFERMERIA

RESULTADOS ESPERADOS	RESULTADOS ESPERADOS

IV EJECUCIÓN

COD	INTERVENCIÓN DE ENFERMERÍA (NIC)	D	N
6550	Protección contra las infecciones, Higiene de manos, mantener normas de asepcia, en señal a la familia medidas de bioseguridad, limpieza terminal y recurrente de la unidad - paciente.		
6840	**Cuidados del niño prematuro:** Regulación de T°, uso de nido - contención, mamá canguro, SNN. estimulación de succión, terapia oral.		
6680	**Monitorización de los signos vitales:** Controlar evaluar T° Ax,FC,FR,PA,(PAM),Sat.02.		
3350	**Monitorización respiratoria:** Evaluar caracteristicas, simetria toraxica, Rx.		
3390	**Ayuda a la ventilación:** Mantener una vía aérea permeable, adm.suplemento de O2 según prescripción, flujo de O2.		
3320	**Oxigenoterapia:** Adm.O2 suplementario por CBN, CPAP, VM vigilar.		
3120	**Intubación y Estabilización de vía aérea artificial:** Verificar posición, altura, TET, cuidados de TET, TQT.		
3180	**Manejo de vías aéreas artificiales:** Cuidados de TET. Prong Nasal, CBN, TQT.		
3160	**Aspiración de las vías aéreas:** Boca, fosas nasales, TET, TQT.		
3300	**Ventilación mecánica:** Monitorear y ajustar parámetros ventilatorios de CPAP, VM, alarmas de VM.		
3270	**Desintubación endotraqueal**		
3310	**Destete de la VM:** Monitoreo parámetros previos asistir al médico.		
3230	**Fisioterapia respiratoria:** Nebulizar (si está indicado) vibración, drenaje postural.		
1872	**Cuidados de drenaje toraxico**		
4250	**Manejo del Shock:** Valorar parámetros hemodinámicos, valores de laboratorio (Hgma, perfil de coagulación, gasometria, cultivo, perfil bioquimico) Adm. vasodilatadores, diuréticos, corticoides, tromboliticos según corresponde.		
1910	**Manejo ácido base:** Obtener muestras, sangre, monitoreo parámetros en VM.		
7690	**Interpretación de datos de laboratorio:** Analizar coherencia con estado clinico del paciente.		
6974	**Resucitación Neonatal:** Realizar RCP avanzada, verificar coche de paro.		
4040	**Cuidados cardiacos:** Monitoreo no cruenta (FR,FC,T°ax. Sat.02) colocar programar monitor no invasivo, valoración global de la circulación periférica (pulso, llenado capilar, color, edema).		
1262	**Monitorización Neurológica:** Vigilar nivel de conciencia, pupilas, reflejos, convulsiones.		
2680	**Manejo de convulsiones:** Valorar caracteristicas de la crisis y mantener FV,		
1400	**Manejo del dolor:** Valoración de escalas, uso SNN, sacarosa uso de analgésico.		
3790	**Inducción a la Hipotermia:** Alcance y mantenimiento de la T° corporal por debajo de 35°C y controlar los efectos secundarios, prevenir complicaciones. Controlar con monitor continuo de T° central. Iniciar enfriamiento externo activo con equipo generador de hipotermia (paquetes de hielo) controlar estado hemodinámica.		
3900	**Regulación de la Temperatura.**		
116	**Monitorización Nutricional:** Manejo de peso, medir perimetro abdominal, admt. alimentación enteral,(cuidado SOG,Uso de jeringa, VO)Eval. tolerancia RIG, nauseas, vomitos, Estimular succión no nutritiva.		
430	**Manejo Intestinal:** Evaluar RH, tensión abdominal, pasaje de deposición y caracteristicas (estimulación enema), valoración del eritema de pañal.		
480	**Cuidado de la Ostomía:** Curación, cambio de gasa vaselinada, apósito, bolsa, uso de pasta-polvo, valoración del eritema periostomal.		
4035	**Toma de Muestras:** Arterial, Venoso, Capilar, fluidos corporales. Valora el resultado.		
590	**Manejo de la eliminación urinaria:** Registrar volum.evaluar gasto urinario, realizar BHE.		

COD	INTERVENCIÓN DE ENFERMERÍA (NIC)	D	N
620	**Cuidados de Retención Urinaria:** Evaluar vol. urinario (gasto urinario) realizar BHE.		
2080	**Manejo de liquidos electrolitos:** Evaluar BHE, valorar Na+, Ca++, SO4Mg.P++.		
6480	**Manejo ambiental:** Manipulación del entorno post beneficios terapeutico, (luz, ruido, manipulación, punción).		
840	**Cambio de posición:** Uso de nido-contensión, posición flexión, línea media - confort.		
3500	**Manejo de presiones:** Prevención de plagiocefalia postural (PP), cambiar posición, rotación según reloj, valorar UPP, rotar sensores.		
1801	**Ayuda con los autocuidados:** Cuidado de piel, limpieza ocular, bucal, genital, curación del cordón umbilical.		
3590	Vigilancia de la piel.		
2240	**Instalación de dispositivos EV:** Vía periférica, cateter percutaneo, cateter umbilical venoso.		
4220	Cuidados de PICC		
2440	**Mantenimiento de Dispositivo de Acceso Venoso:** PICC, CVU, CAU, Cateter periférico siguiendo protocolo de servicio.		
1200	**Administración de NTP:** Mantener técnica esteril y precauciones universales al instalar las soluciones, vigilar signos anormales, efectos adversos.		
2300	**Administración de medicación:** Seguir 5 principios en TTO, enteral (VO, SOG) parenteral (IM, SC, IV), inhalación, oftálmico, tópico, rectal, inhalación, NBZ. Valorar efectos adversos.		
2210	**Administración de analgésicos:** Sedantes, registrar rpta. y cualquier efecto adverso.		
4030	**Administración productos Sanguineos:** Paquete globular, Plasma, Crioprecipitado Plaquetas Sangre total (ETT)		
4160	**Control de Hemorragia:** Identificar, observar, aplicar presión sobre el punto de hemorrágico, evaluar respuesta.		
2880	**Coordinación preoperatoria:** Revisar y confirmar programación, consentimiento informado, aporte hidrico EV, coordinación con enfermera quirúrgica.		
2870	**Cuidado Post Anestesia:** Obtener información de la enfermera quirúrgica y del anestesista, valorar signos vitales, estado neurológico, nivel de conciencia, asegurar termorrgulación, diuresis, valorar analgesia.		
6540	**Control de Infecciones:** Cambiar set. Corrugados (VM, CPAP, CBN, Neopuff), barboteadores, set de infusión IV, Sonda Vesical, SOG Según protocolo del servicio.		
6924	**Fototerapia del Neonato:** Selección de equipo, protección ocular, gonadas, hidratación, vigilar luces, distancia de fototerapia.		
7140	**Apoyo a la familia:** Valorar, ofrecer, escuchar asegurar que se brinde los mejores cuidados posibles.		
5244	**Asesoramiento de Lactancia:** Proporcionar, ofrecer, corregir, evaluar para asegurar el mantenimiento de una alimentación de pecho satisfactoria, extracción precoz del calostro para inicio del NEM.		
7370	**Planificación de alta:** Coordinar, comunicar con el siguiente nivel de cuidados.		
960	**Transporte:** Transferir, usar incubadora equipada, equipo de reanimación.		
6630	**Aislamiento:** Determinar necesidad, tipo de aislamiento, restricción de ingresos, uso de EPR supervisar la limpieza, desinfección del entorno del paciente.		
5260	**Cuidados de la Agonía:** Proporcionar intimidad, facilitar apoyo espiritual a la familia.		
1770	**Cuidados post Mortem.**		
7920	**Documentación:** Llenado de formatos.		

V. EVALUACIÓN: RESULTADOS OBSERVADOS

FIRMA Y SELLO

FIRMA Y SELLO
